# Supplementary material for: The Hidden Secrets of the Dental Calculus: Calibration of a Mass Spectrometry Protocol for Dental Calculus Protein Analysis
Source: Int J Mol Sci. 2022 Nov 19;23(22):14387. doi: 10.3390/ijms232214387 (PMC9698480; doi:10.3390/ijms232214387)
Supplement: Supplementary file 1 [file ijms-23-14387-s001.zip › ijms-2009557-Supplementary File S1.pdf]

Washes

1. Wash sample with 500 µL PBS for 15 min in a 1.7ml tube in a 3D orbital shaker.
2. Centrifuge for 10 min at 10000 RCF and remove the supernatant.
3. Repeat the wash and centrifuge (1-2) 3 times.

Demineralization

4. Add 500 µLl of 25% Acetic acid in DDW, leave overnight in a 3D orbital shaker.
5. Crush the calculus with micropestels.
6. Centrifuge for 10 min, at 4°C and 10,000 RCF.
7. Remove the Acetic acid, add 300 µL PBS, and vortex (up and down).
8. Repeat the washes 4 times.
9. After the last centrifuge replace the PBS with 150 µL lysis buffer (pH 7.5: 2M Guanidine Hydrochloride (Merck), 10mM Chloroacetamide (Bar Naor - Israel), and 5mM HEPES (Merck).
10. Sonication for 25 min at 40% (Sonic Ruptor 400, Omni).
11. After sonication, spin down for 30 min at 4°C at 21,000 RCF.
12. Separate the pellet from the supernatant, keep supernatant.

Precipitation

Aceton (recommended)

13. Add 600 µL of cold (-20°C) Acetone (equal to 4 times the sample volume) and vortex thoroughly.
14. Put the sample at -80°C for 60 min.
15. Centrifuge at 15,000 RCF for 10 min at 4°C, discard Acetone while keeping the pellet.
16. Using a speedvac or a desiccator, completely remove acetone remains.
17. Resuspend with 100 µL of 100mM HEPES.

Methanol-Chloroform (alternative)

18. For a sample volume of 150 µL add 600 µL Methanol and vortex.
19. Add equal amounts of Chloroform to the original sample (150 µL) and vortex.
20. Add 400 µL of double distilled water and vortex thoroughly, until the mixture is cloudy with precipitation.
21. Centrifuge at 14,000 RCF for 1 min.
22. Remove the top layer without disturbing the circular flake of protein.
23. Add Methanol equal to 4 times the sample volume and vortex.
24. Centrifuge for 5 min at 20,000 RCF.
25. Discard excess Methanol.
26. Lyophilize the sample overnight.
27. Resuspend with 100 µL of 100mM HEPES.

Reduction

28. Add Dithiothreitol to a final concentration of 10mM.
29. Incubate in a 3D orbital shaker for 60 min at 55°C and 200 RPM.

Alkylation

30. Immediately before use, add 100 mM HEPES to Iodoacetamide to a final concentration of 18.75mM Iodiacetamide.
31. Incubate for 30 min at room temperature protected from light**.**

Trypsinization

32. Add 10nG mass spectrometry grade Trypsin (2 uL of 5 nG / uL stock).
33. Incubate overnight at 37°C and 200 RPM.
34. After overnight digestion, stop trypsinization by adding 1μL of Trifluoroacetic acid (TFA 0.1%).

Solid Phase Extraction Stage Tips for Detergent Removal

C18 Empore™ Stage - Tip

35. Use 2 layers of C18 Empore™ SPE disks, punched and inserted into 200 μL tips.
36. Activate with 100 μL MeOH.
37. Centrifuge for 2 min at 1500 RCF and discard excess fluids.
38. Activate and clean from residual peptides with 100 μL buffer B (80% acetonitrile,0.1% TFA)
39. Centrifuge for 2 min at 1500 RCF and discard excess fluids.
40. Return to hydrophilic state with 100 μL buffer A (0.1%TFA).
41. Load up to 6 μG protein.
42. Centrifuge for 2 min at 1500 RCF and discard excess fluids.
43. Return to hydrophilic state with 100 μL buffer A (0.1%TFA)
44. Repeat the previous step.
45. Manually elute with 60 μL of buffer B.
46. Dry the elution tube under a vacuum using a speedvac or a lyophilizer.

SCX strong cation exchange Empore™ Stage Tip:

47. Use 2 layers of C18 Empore™ SPE disks.
48. Wash the layers with 400 μL of TFA 0.1%.
49. Resuspend the sample with 100 μL TFA 0.1%.
50. Load the sample and pass it through manually.
51. Wash with 200 μL TFA 0.1%.
52. Elution with 100 μL methanol 30% ammonium hydroxide 5%.
53. Dry the elution tube under a vacuum using a speedvac or a lyophilizer.

Liquid Chromatography and Mass Spectrometry:

54. Resolve peptides by reverse phase chromatography on 0.075 X 180 mm fused silica capillaries (J&W) packed with Reprosil reverse phase material C18 (Dr Maisch GmbH, Germany).

55. Elute peptides with either 60 minutes or 120 minutes linear gradient of 5% to 28% 15 minutes gradient of 28 to 95% and 25 minutes at 95% Acetonitrile with 0.1% Formic acid in water at flow rates of 0.15 µl/min.
56. Perform mass spectrometry by Q Exactive HF mass spectrometer (Thermo) in a positive mode using repetitively full MS scan followed by collision-induced dissociation of the 18 most dominant ions (>1 charge) selected from the first MS scan.
57. Perform a dynamic exclusion list with an exclusion duration of 20 seconds.
